# P-1137. Molecular and Culture-Based Surveillance of Environmental Clostridioides difficile Contamination

**DOI:** 10.1093/ofid/ofaf695.1331

**Published:** 2026-01-11

**Authors:** Amanda M Graves, Guerbine Fils-Aime, Aaron Barrett, Becky A Smith, Nicholas A Turner, Deverick J Anderson, Bobby G Warren

**Affiliations:** Duke University School of Medicine Duke Center for Antimicrobial Stewardship and Infection Prevention, Durham, NC; Duke School of Medicine, Durham, North Carolina; Duke Health, Cary, North Carolina; Duke University, durham, North Carolina; Duke University Medical Center, Durham, NC; Duke Center for Antimicrobial Stewardship and Infection Prevention, Durham, NC; Duke University School of Medicine, Hillsborough, North Carolina

## Abstract

**Background:**

Environmental contamination by *Clostridioides difficile* poses a significant challenge in healthcare settings. Molecular detection was performed using a C. difficile-specific16S rRNA primer alongside the tcdB toxin gene target. We compared molecular detection (16S and *tcdB* gene qPCR) and culture positivity.

**Table 1.**
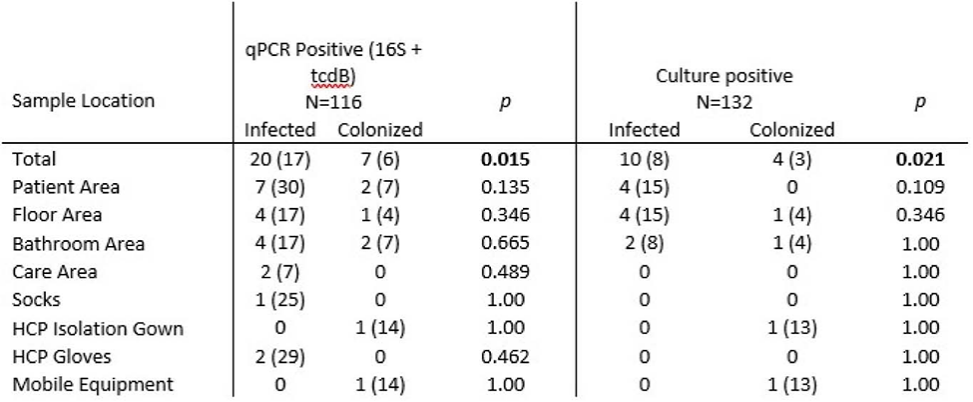
Molecular and Culture Positivity by Sample Area and Patient Infection Status

**Figure:**
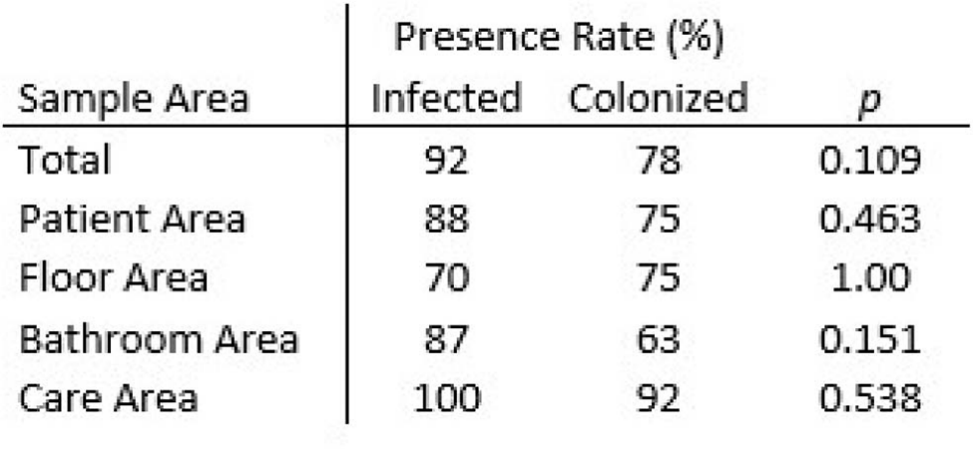
Fluorescent Marker Presence by Sample Area and Patient Infection Status

**Methods:**

We conducted a prospective study to measure environmental contamination using molecular and culture-based detection methods, grouping by infection status. All enrolled patients (N=10) were C. difficile PCR-positive; 5 were toxin-positive (defined as infected), and 5 were toxin-negative (defined as colonized). Samples were tested for C. difficile 16S rRNA and *tcdB* via qPCR and cultured on selective media. A sample was considered qPCR-positive if both targets were detected. Additionally, fluorescent markers were applied to predefined high-touch surfaces in each patient room to assess environmental cleaning.

**Results:**

A total of 132 environmental samples were collected in 10 patient rooms from 7 predefined zones: patient area (bedrails, overbed table, footboard), bathroom area (toilet seat, bathroom sink, floor around the toilet), floor area (between the patient bed and bathroom), care area (computer keyboard, mouse, IV pole), socks, mobile equipment, and healthcare providers’ PPE (isolation gown and gloves). Of 116 samples tested by qPCR, 20 (17%) were positive for both 16S and *tcdB* from environments of infected patients, and 7 (6%) from colonized patients (p=0.021) (Table 1). Of 132 samples, 10 (8%) were recovered via culture C. difficile from infected patients and 4 (3%) from colonized patients (p=0.015). The highest positivity rates were observed in patient areas, bathrooms, and floors, with minimal detection observed on HCP PPE and mobile equipment. Fluorescent markers were observed in rooms of infected (92%) and colonized patients (78%), suggesting no substantial difference in environmental cleaning between groups (Table 2).

**Conclusion:**

In this cohort of PCR-positive patients, molecular detection consistently identified more environmental contamination than culture, with the greatest burden in environments of infection. Similarly, culture positivity was significantly higher in environments of infected patients compared to colonized patients.

**Disclosures:**

Nicholas A. Turner, MD, MHSc, PDI: Grant/Research Support|Purio Labs: Grant/Research Support

